# Risky Sexual Behavior and Associated Factors among Street Youth in Dilla Town, Gedeo Zone, South Ethiopia, 2018

**DOI:** 10.4314/ejhs.v31i5.5

**Published:** 2021-09

**Authors:** Simret Fikre, Girma Tenkolu, Zerihun Berhanu Mamo

**Affiliations:** 1 Student Clinic, Dilla University, Dilla, Ethiopia; 2 Department of Public Health, School of Public Health, College of Health Sciences and Medicine, Dilla University, Dilla, Ethiopia; 3 Department of Reproductive Health, School of Public Health, College of Health Sciences and Medicine, Dilla University, Dilla, Ethiopia

**Keywords:** Risky Sexual Behavior, Street Youth, Ethiopia

## Abstract

**Background:**

Street youth are exposed to situations that make them vulnerable to sexual and reproductive health problems. The majority of street youth are living in conditions of severe deprivation, which place them at all kinds of health risks. Street youth have risky sexual behaviors that increase the likelihood of adverse sexual and reproductive health consequences. Therefore, this study aimed to identify the prevalence and associated factors of risky sexual behavior among street youth in Dilla town, Gedeo zone, South Ethiopia, 2018.

**Methods:**

A cross-sectional study was conducted among 279randomly selected street youth after locating and identifying them through census using a structured pre-tested questionnaire. Descriptive and binary logistic regression analyses were used. Statistically significant was declared at alpha<0.05.

**Results:**

The prevalence of risky sexual behavior among street youth in Dilla town was53.9% (95% CI -(48, 60.2)). Female sex (AOR=9.57, 95% CI- (1.76, 52.07)), age (AOR=1.23, 95% CI-(1.08, 1.39)), educational level (AOR=3.00, 95% CI- (1.08, 8.33)) and alcohol intake (AOR=2.27, 95% CI – (1.11, 4.68)) were statistically significant with risky sexual behavior.

**Conclusion:**

A substantial number of street youths were engagedin risky sexual behavior, while female sex, increase in age, educational level, and alcohol intake of street youth were found to contribute to aggravate the problem. This calls formobilizing interventions considering the above factors to bring behavioral change in reducing risky sexual practices.

## Introduction

More than half of the world's population is below the age of 25 years, and four out of five young people live in developing countries. Sub-Saharan Africa has one of the world's youngest populations. In Ethiopia, the sexual and reproductive health of young people has become a major public health concern due to a high prevalence of sexual transmitted infections (STIs) like HIV/AIDS among young people. It is estimated that young people age 10–24 years constitute more than a third of the population, 26.5 million (33%) (1).

Ethiopia is one of the countries where child with street life is high. The extent and nature of street children is one of the most serious social problems in urban areas of Ethiopia and become a countrywide problem today(2). Thus, there are a large number of children living and/or working on the streets of different cities of the country.

Even though there is no published data, various reports from zonal Labor and Social Affairs Division indicate a rapid increase of street children in many parts of Southern Nation, Nationalities and Peoples' Region(SNNPR), Hawassa, Wolkete, Dilla and Wollayta Soddo are the major towns of the region encountering a growing number of street children (3).

Street youth are exposed to situations that make them vulnerable to sexual and reproductive health problems on a day to day basis. A risky sexual behavior is one that increases the likelihood of adverse sexual and reproductive health consequences (4).

Risk sexual behavior is a behavior related to sexuality which increases the susceptibility of an individual to problems related to sexuality and reproductive health like sexually transmitted disease (STIs), human immune deficiency virus (HIV), unwanted and unplanned pregnancy, abortion, and psychological distress (5). Risk sexual behavior includes having more than one sexual partner, early sexual initiation, inconsistent use of condom, and having sex with commercial sex workers (6). Therefore, this study intended to identify the prevalence and associated factors of risky sexual behavior among street youth in Dilla town, Gedeo zone, South Ethiopia.

## Materials and Methods

**Study design and area**: A community-based cross-sectional study was employed from March 19- May 13/2018. It was conducted in Dilla Town, Gedeo Zone, South Nation Nationality and Peoples Region which is located 360km away from Addis Ababa, the capital city of Ethiopia, and 90 km away from the administration center of SNNPR (Hawassa). Dilla town administration has 03 sub-cities, which includes 09 kebeles. It has a total population of 91,534 from these 36,613 are youth (source; Gedeo zone youth office) and according to the survey conducted 784 youths are living in the street of the city.

**Population**: The source population was all street youth aged 10–24 years residing in Dilla town. The study population was randomly selected street youth who have resided in Dilla town for at least six months. All street youth who are seriously ill and unable to hear were excluded from the study.

**Sample size determination**: The minimum required sample size was calculated using single population proportion formula assuming 43.1% on risky sexual behavior among youth according to a study from Addis Ababa(7), 5% margin of error, and 95% confidence level. The final sample size was 279 (including a 10% non-response rate).

**Sampling procedure**: To develop the sampling frame, the census was done for two days during the night time resulting in784street youths in the town. Then the study subjects were selected by a simple random sampling method from the list of a sampling frame.

**The following operational definitions were used for this study**

**Risky sexual behaviors (practice**): In this study it is defined as one of the following: never use of condoms or inconsistent use of condoms, having more than one sexual partner or starting sex before age 18 years or having sex with sex workers/ commercial sex worker.

**Substance abuse**: Youth, who drink alcohol, chew chat, smoke cigarette, and sniff benzene regardless of the amount and frequency of use.

**Street youth**: In this study, it is defined as homeless people between age 10 and 24 years living on the street and had no fixed place of residence.

Data collection procedures: The data were collected by using an interviewer-administered questionnaire adapted and modified after reviewing different related literature as appropriate to address the study objectives. The questionnaire was prepared originally in English and then translate in to Amharic and back to English. It was administered to the respondents in Amharic to make the information easily understood by the data collectors and respondents during data collection and to get consistent information. Participants were asked if they had already given a similar interview in the two or three preceding days. A selected study site was visited only once.

Eight data collectors (who were diploma nurses) and two supervisors were recruited and training on the objective of the study and techniques of data collection for one day was given. Pre-testing of the questionnaire was done in 5% (14 participants) of the sample in the Dilla zuria district.

To assure the data quality high emphasis was given in designing a data collection instrument. Before starting the actual survey, the questionnaire was pre-tested on 5% of the sample. During the pre-test, the questionnaire was assessed for its consistency, clarity, understandability, completeness, reliability.

The quality, validity, and reliability of data had been assured through, careful design, translation and retranslation of the tool and proper training of the data collector and supervisors, close supervision of the data collectors, and proper handling of the data. Data collection was also monitored frequently in the field and the collected data had been reviewed and checked for completeness and consistency before data entry.

**Data analysis procedures**: The data was entered into EPI data version 3.5.1 and then exported to SPSS version 20 statistical program for analysis. Descriptive statistics like frequency, percentage, mean and standard deviation had been conducted to describe the data by examining and summarizing the distribution of each variable.

Binary logistic regression has been done to identify associated factors with the outcome variable. Candidate variables for multivariable analysis were identified through bivariable analysis at alpha<0.25 and multicollinearity (r<0.5). Odds ratio (OR) and confidence interval (CI) at 95% confidence level were used for decision and the level of significance was declared at alpha< 0.05. Hosimer Lemshow test (0.866) was used to fit the overall model. Text and table were used to present the finding of the study.

**Ethics approval and informed consent**: Ethical clearance letter to conduct this study was obtained from Dilla University, institutional review board then submitted to zonal Labor and Social Affairs Division, and city administration. Also, assent was obtained from each study participant who was agreed to participate by informing and discussing the purpose of the study (As the standard practice with self-reliant youth populations (< 18yrs) not in contact with or otherwise supervised by an adult guardian, the researchers considered this as an appropriate means to seek informed consent from the participants). Participants were assured of their profile and the response they would give not exposed to anyone in any circumstances.

## Results

A total of 279 street youth interviewed, of which 10 respondents were excluded for gross incomplete and inconsistent responses making the response rate 96.4%. The analysis was made based on the 269 completed questionnaires.

**Socio-demographic characteristics**: Of the total study participants, 248 (92.2%) were males. The mean age was 16.56 (SD± 3.09) years while more than half of street youth were between the age of 15 and 19 years. Most participants, 154 (56.9%) reported that they were educated up to grade 8. Around two-fifths of the participant were engaged in carrying small items. While shoe shining, exchange of money for sex, and peddling were the means of survival for few participants and half of them 139(51.70 %) earn on average 10 to20 birr per day (Table 1).

**Table 1:** Socio-demographic characteristics of street youth in Dilla town, Gedeo zone, South Ethiopia, 2018 (n=269)

Variables	Frequency	Percent
**Sex**		
Male	248	92.2
Female	21	7.8
**Age group** (in years)		
10–14	76	28.3
15–19	141	52.4
20–24	52	19.3
**Religion**		
Orthodox	89	33.1
Muslim	16	5.9
Protestant	101	37.5
Catholic	7	2.6
No religion	56	20.8
**Educational level**		
Unable to read and write	76	28.3
Read and write	34	12.6
Grade 1–8	159	59.1
**Work to earn money**		
Yes	256	95.2
No	13	4.8
**Work type to earn money**		
Carrying items	106	39.4
Transferring messages	79	29.4
Begging	63	23.4
Other+	21	7.8
**Average income per day**		
<10 birr	109	40.5
10–20 birr	139	51.7
>20 birr	21	7.8
**Former of residence**		
Dilla	99	36.8
Out of Dilla	170	63.2
**Duration of stay on the street**		
<6 month	21	7.8
6 – 12month	93	34.6
1 – 3 years	96	35.7
More than 3 years	59	21.9
**Sleeping area during the night**		
On the street	242	90.0
Plastic shelter	27	10.0
**Substance intake**		
Alcohol	188	69.9
Cigarette	119	44.2
Chat	101	37.5
Benzene	113	42.0

+ Includes car washing, exchanging money for sex, shoe shining and peddling

**Reproductive and sexual health characteristics**: The overall prevalence of risky sexual behavior was 53.9% (95% CI – (48, 60.2)). While 151 (56.1%) respondents reported as they had experienced sexual intercourse in their lifetime. The mean age at sexual commencement was found to be 15.7 years (SD ±2.3). Among sexually active study participants, 120 (79.5%) started sexual intercourse earlier than 18 years of age. Among youth who had been sexually active, 82 (54.3%) had more than one lifetime sexual partners. Hundred twelve (74.2%) of street youths had sexual intercourse at least once in the past twelvemonths (Table 2).

**Table 2:** Reproductive and sexual health characteristics of street youth in Dilla town, Gedeo zone, South Ethiopia, 2018 (n=269)

Variables	Frequency	Percent
**Risky sexual behavior**		
Yes	145	53.9
No	124	46.1
**Ever had sexual intercourse**		
Yes	151	56.1
No	118	43.9
**Age at first sexual intercourse n=151**		
<18	120	79.5
≥18	31	20.5
**With whom did you make your first sexual intercourse n=151**		
With a casual boy/girl friend	75	49.7
With a steady boy/girl friend	39	25.8
With a family member	12	7.9
With commercial sex worker	25	16.6
**Life time number of sexual partner**		
One	69	45.7
Two and above	82	54.3
**Have you had sexual intercourse in the past 12 months n=151**		
Yes	112	74.2
No	39	25.8
**With how many partners have you had sexual intercourse in the past 12 months**		
One	57	50.9
Two and above	55	49.1
**Ever use of condoms**		
Yes	83	55
No	68	45
**Condoms use during the first sexual intercourse**		
Yes	31	20.5
No	120	79.5
**Sex with commercial sex worker** n=96		
Yes	40	41.7
No	56	58.3
**Condom used during sexual intercourse with commercial sex worker**		
Yes	26	65
No	14	35
**Sex after alcohol intake**		
Yes	73	48.3
No	78	51.7

**Factors associated with risky sexual behavior**: Sex, age, educational status, work type, average daily income, previous residence, length of stay on the street, type of sleeping place, alcohol drinking, cigarette smoking, and benzene sniffing were candidate variable for multivariable binary logistic regression after passing bi-variable binary logistic regression (α<0.25) and multicollinearity (r<0.5). Sex, age, educational status, and alcohol intake were statistically significant factors associated with risky sexual behavior among street youth after controlling for work type, average daily income, previous residence, length of stay on the street, type of sleeping place, cigarette smoking, and benzene sniffing.

Female street youth had 9.6 times more likely hood to engage in risky sexual behavior than male street youth (AOR=9.57, 95% CI- (1.76, 52.07)). The odds of risky sexual behavior increase by 1.2 for a unit increase in the age of street youth (AOR=1.23, 95% CI- (1.08, 1.39)). As per the educational level; those street youth who read and write were three times more likely to have risky sexual behavior than those whom unable to read and write (AOR=3.00, 95% CI-(1.08, 8.33)). Also, street youth with alcohol drinking experience had 2.3 times more likely hood of engaging in risky sexual behavior than their counterpart (AOR=2.27, 95% CI – (1.11, 4.68)) (Table 3).

**Table 3:** Associated factors with risky sexual behavior of street youth in Dilla town, Gedeo zone, South Ethiopia, 2018

Variables	Risky sexual behavior		COR (95% CI)	AOR (95% CI)
	No (%)	Yes (%)		
Sex				
Male	122(98.4)	126(86.9)	1	1
Female	2(1.6)	19(13.1)	9.2(2.1, 40.33)**	9.57(1.76, 52.07)**
Age			1.33(1.20, 1.46)**	1.23(1.08, 1.39)**
Educational level				
Unable to read and write	47(37.9)	29(20)	1	1
Read and write	9(7.3)	25(17.2)	4.50(1.85, 10.98)**	3.00(1.08, 8.33)*
Primary(grade 1–8)	68(54.8)	91(62.8)	2.17(1.24, 3.80)**	1.29(0.62, 2.67)
Alcohol drinking				
No	55(44.4)	26(17.9)	1	1
Yes	69(55.6)	119(82.1)	3.65(2.10, 6.34)**	2.27(1.11, 4.68)*

* α<0.05

** α<0.01

Hosimer Lemshow test (α= 0.866)

## Discussion

In this study, the prevalence of risky sexual behavior among street youth was 53.9% (95% CI – (48, 60.2)) whereas sex, age, educational level, and alcohol intake of street youth were associated factors with their risky sexual behavior.

The study revealed that more than half of street youth were engaged in risky sexual behavior which is comparable with a study from Kenya (8) whereas, it was a bit higher than that found among school youth in Sri Lanka(9), Nigeria(10) and Ethiopia (7, 11–21). This indicated that street youth had higher risky sexual behavior when compared with non-street youth which could be explained by being on street exposes them to early sexual activity and higher prevalence of substance abuse and lack of access to information. This finding is lower than the study conducted among street children in Addis Ababa (22) and Dessie (4); and among non-street youth in Nekemte (23), Tigray (24) and Gondar (1) of Ethiopia. This variation could be due to the age group difference of the study participants as this study included younger age groups of 10–24 years old as compared with older age groups of 15–24 years. Another possible reason for the difference might be because of the small sample size used in this study.

According to this study being female was found 9.6 times more likely to have risky sexual behavior than their counterparts. A similar finding was reported in a study conducted in Gondar (1) and Mekelle (25). Life on the street would be riskier for females than males exposing them to different sexual exploitation and may be expected to be engaged in risky sexual activities to survive. In contrast studies from the Bahamas (26), Kenya (8) and Ethiopia (14, 17, 24) revealed as males had more risky sexual behavior. The alteration may be attributed to variation in the population.

The odds of risky sexual behavior increase by 23% for a unit increasing in the age of street youth. A comparable finding was reported in studies from Sri Lanka (9), Bahamas (26), Kinshasa (27), Gondar (1), Nekemte (23) and South Ethiopia (17). It might be due to that older street children, those who have been longer on the streets, and tend to use drugs more, which increases their likelihood of being involved in risky behavior.

The study also identified a negative association between educational level and risky sexual behavior, in which those street youth who were able to read and write were 3 times more likely to exercise risky sexual behavior than those street youth who were unable to read and write. It was supported by a finding from Tigray Ethiopia(24). Despite little knowledge, youth continue to experiment with high-risk sexual behaviors. This might be due to low-risk perception and lack of sustainable education on this issue, which predisposes youth to high-risk sexual activity as supported by this study. But it is in contrast to an adult study in Addis Ababa (11). The difference may be due to the population and set up.

Alcohol consumption was another factor found to increase risky sexual behavior among street youth in Dilla. This was in line with the study finding from Sri Lanka(9) and Ethiopia (1, 17, 24). Substance use like alcohol could contribute to risky behavior development and engagement.

A face-to-face interview was used which may not be convenient to study sexuality in conservative societies. Also, the study topic by itself assesses personal and sensitive issues related to sexuality which might have caused underreporting of some behaviors. Thus, the finding of this study should be interpreted with these limitations.

In conclusion a substantial number of street youths were engagedin risky sexual behavior, while female sex, increase in age, educational level, and alcohol intake of street youth were found to contribute to aggravate the problem. This fact calls for a coordinated and comprehensive effort by responsible organisations to mobilise interventions considering the above factors to bring behavioral change in reducing risky sexual practices.
